# Exploring the Mechanisms of Total Saponins of Black Ginseng and Ginsenoside Rg3 Against Doxorubicin‐Induced Cardiotoxicity

**DOI:** 10.1002/fsn3.71968

**Published:** 2026-06-08

**Authors:** Linlin Liu, Peiyuan Dou, Xiaotong Zhang, Xiaoku Ran, Deqiang Dou, Xiaodong Lv

**Affiliations:** ^1^ Liaoning University of Traditional Chinese Medicine Dalian China; ^2^ Tianjin University of Traditional Chinese Medicine Tianjin China

**Keywords:** black ginseng, DOX‐induced cardiotoxicity, doxorubicin, ginsenoside Rg3, HDAC8/BRCA2/DRP1

## Abstract

Doxorubicin (DOX), a widely utilized chemotherapeutic agent, is constrained by its intricate cardiotoxicity, which involves oxidative stress, mitochondrial dysfunction, and cell death. Black ginseng (BG), processed to enhance rare saponins such as ginsenoside Rg3, demonstrates augmented anticancer and cardioprotective properties. The combination of DOX with total saponins of BG (TSF) or the active component Rg3 may provide synergistic antitumor effects while mitigating cardiotoxicity, although the underlying mechanisms necessitate further investigation. This study aimed to evaluate the protective effects of TSF and Rg3 against DOX‐induced cardiotoxicity (DIC) and to explore their potential to enhance mitochondrial function by modulating the HDAC8/BRCA2/DRP1 pathway. The research involved in vitro studies using H9c2 cells and in vivo experiments with mouse models to assess the pharmacological actions of TSF and Rg3. Network pharmacology was employed to predict the potential mechanisms by which TSF regulates the HDAC8/BRCA2/DRP1 pathway. Verification was conducted through Western blot analysis and immunofluorescence colocalization techniques. In vitro experiments demonstrated that TSF and Rg3 enhanced the viability of H9C2 cells subjected to DOX induction. In vivo studies revealed that TSF and Rg3 significantly improved cardiac function and mitigated myocardial structural damage in mice. Notably, the effects of Rg3 were particularly pronounced. TSF and Rg3 also effectively reduced oxidative stress markers, such as SOD and MDA, and decreased inflammation. Furthermore, they inhibited apoptosis, restored cardiomyocyte morphology, and enhanced mitochondrial function by increasing ATP activity. Cyberpharmacological analysis suggested that the HDAC8/BRCA2/DRP1 signaling pathway may be a target for regulation by TSF and Rg3 during DOX‐induced progression. Our findings demonstrated that TSF and Rg3 reverse DOX‐induced upregulation of HDAC8 and DRP1 protein expression while downregulating BRCA2 protein expression in cardiac tissues. Additionally, immunofluorescence colocalization confirmed direct interactions among HDAC8, BRCA2, and DRP1. TSF and Rg3 demonstrate significant cardioprotective effects against DIC by mitigating oxidative stress, inflammation, and apoptosis, while preserving mitochondrial function. These findings provide novel insights into the clinical relevance of TSF and Rg3 in the management of DIC. The regulation of the HDAC8/BRCA2/DRP1 pathway underscores the potential of TSF and Rg3 as a promising therapeutic strategy for the prevention and treatment of DIC.

## Graphical abstract

TSF and Rg3 demonstrate significant cardioprotective effects against DOX‐induced cardiotoxicity (DIC) by mitigating oxidative stress, inflammation, and apoptosis while preserving mitochondrial function. These findings provide novel insights into the clinical relevance of TSF and Rg3 in the management of DIC.

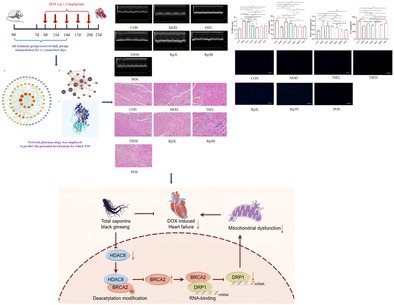

## 1 Introduction

With the continuous progress of tumor treatment, the number of tumor survivors is increasing, but tumor‐induced cardiovascular diseases, especially the cardiotoxicity caused by antitumor therapy, seriously affect the prognosis of tumor patients and become a new challenge for both oncologists and cardiologists (Austin et al. 2024).

Doxorubicin (DOX), a widely used broad‐spectrum antitumor drug in clinical settings, is frequently associated with chemotherapy‐induced cardiotoxicity (Qiao et al. 2024). However, its clinical application has been hampered by cardiotoxicity. DOX‐induced cardiotoxicity (DIC) is complex, potentially involving interconnected processes such as reactive oxygen species (ROS) production, mitochondrial dysfunction, cell membrane damage, apoptosis, and calcium overload (Li et al. 2017; Scott et al. 2011; Zhang et al. 2012). Researchers have focused on screening and identifying drugs to reduce cardiotoxicity.

The roots and rhizomes of *Panax ginseng* C. A. Mey., widely recognized as ginseng (GS), are globally renowned medicines. Black ginseng (BG) is a newly developed ginseng product that enhances rare saponins or secondary ginsenosides through a repeated nine‐step steaming and drying process. Our research group previously discovered that BG exhibits superior anticancer and anti‐heart failure (anti‐HF) effects compared to GS and red ginseng (RG), due to its higher levels of rare saponins (Dou et al. 2024; Liu, Han, et al. 2024; Liu, Jin, et al. 2024; Zhu et al. 2019). Among the bioactive components of BG, ginsenoside Rg3 stands out as a major active constituent with diverse pharmacological effects. Emerging evidence indicates that Rg3 not only exerts notable cardioprotective effects but also possesses potent antitumor activity (Huang et al. 2023; Zhang et al. 2024). These findings suggest that combining DOX with either total ginsenosides of BG (TSF) or Rg3 could potentially enhance antitumor efficacy while mitigating DIC through synergistic mechanisms. The exact underlying mechanisms have yet to be completely understood.

Network pharmacology is a novel strategy for systematically investigating the relationships among diseases, drugs, and targets. Growing evidence highlights its effectiveness in clarifying the molecular mechanisms of herbs with complex multi‐compounds (Chung et al. 2023). This study utilized a network pharmacology approach to systematically identify potential targets of BG ginsenosides. Building upon this foundation, we integrated literature mining and bioinformatics analysis to predict key downstream proteins and their mechanisms of action in alleviating DIC. The predicted mechanisms were validated through pharmacodynamic studies conducted both in vitro and in vivo. This integrated strategy offers a scientific foundation for the potential clinical use of total ginsenosides of BG (TSF) and Rg3.

## 2 Materials and Methods

### 2.1 Key Materials

TSF were isolated from BG extract by our laboratory and were identified by HPLC (For detailed information please see Supporting Information S1). While kits for Creatine kinase‐MB (CK‐MB), superoxide dismutase (SOD), malonaldehyde (MDA), and Adeno‐sine 5 triphosphate (ATP) were procured from Nanjing Jiancheng Bioengineering Institute, China (Lot: 20240925、20240927、20241009、20240927). The Anti‐HDAC8 Rabbit was procured from Solarbio Science & Technology Co. Ltd., Beijing, China (Lot: AC241204061). Antibodies for BRCA2, DRP1 and Bcl‐2 were sourced from Wanlei Biotechnology Co. Ltd. (Lot: T11052438, T09113028, T11201556), while *β*‐actin and HRP‐labeled secondary antibodies were procured from Affinity Biosciences (Lot: T0022, S0001).

### 2.2 Network Pharmacology Investigates TSF’ Mechanism Ameliorate DOX‐Induced Myocardial Injury

First, Ginsenosides of BG and their putative human targets were identified using the TCMIP v2.0, supplemented by target prediction via PubChem and Swiss Target Prediction databases. Secondly, HF‐related targets were obtained from the GeneCards, OMIM, and TTD databases. The intersection between these disease targets and the drug targets was determined using the Venny online tool. Subsequently, a “drug‐component‐disease‐target” interaction network was constructed and analyzed using CytoScape software. The intersecting targets were imported into the STRING database to create a protein–protein interaction (PPI) network. This PPI network was further analyzed in CytoScape to identify core targets, followed by molecular docking verification. Additionally, to explore potential regulatory mechanisms, posttranslational modification information for the BRCA2 protein was queried from the PhosphoSitePlus database, and the binding tendency between BRCA2 protein and DRP1 mRNA was predicted using the RNAct database.

### 2.3 Animal Experiments

**FIGURE 1:**
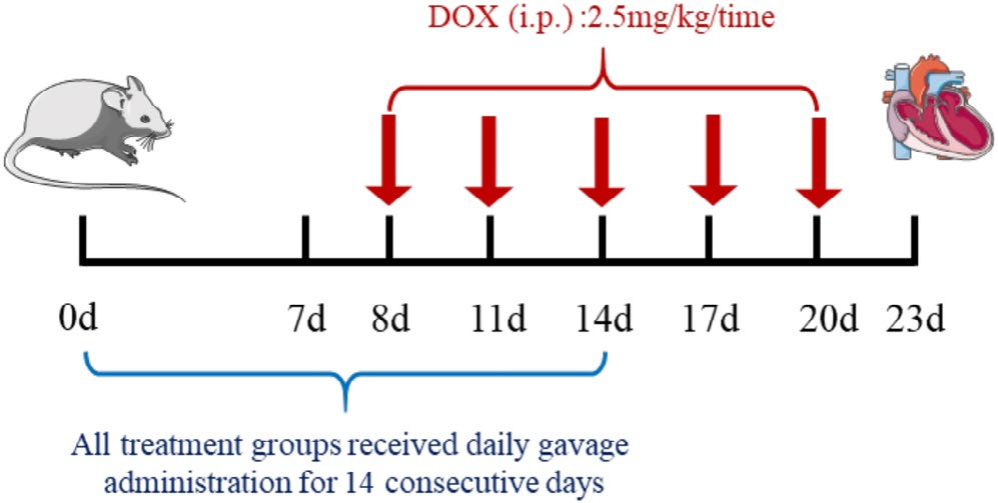
Experimental timeline.

Male C57BL/6 mice, aged 8–10 weeks and weighing 18–22 g, were obtained from Liaoning Changsheng Biological Co. LTD.: SCXK (Liao): 2023–0001. Males were selected for the study to avoid the potential cardiac protective effects of estrogen and higher telomerase activity in females, which may enhance tissue regeneration capacity (Childs et al. 2002; Kang et al. 1997). Mice were housed in pathogen‐free conditions with a 12‐h light/dark cycle at 25°C. All procedures adhered to the guidelines of the Liaoning University of Traditional Chinese Medicine Institutional Animal Care and Use Committee. The Liaoning University of Traditional Chinese Medicine Institutional Animal Care and Use Committee approved this research (No. 21000062024101. Date: September 2, 2024). After a 1‐week acclimation period, mice were randomly divided into seven groups: Control (CON), Model (MOD), DOX with low‐dose TSF (TSFL, 3.13 × 10^−2^ g/kg), DOX with high‐dose TSF (TSFH, 6.25 × 10^−2^ g/kg), DOX with low‐dose Rg3 (Rg3L, 5.2 mg/kg), DOX with high‐dose Rg3 (Rg3H, 15.6 mg/kg), and Enalapril (POS, 2.6 mg/kg). All treatment groups received daily gavage administration for 14 consecutive days, while the CON and MOD groups were administered an equal volume of distilled water (4 mL/kg) via gavage. On Day 8, 30 min after gavage, all groups except CON were administered intraperitoneal injections of DOX (2.5 mg/kg) every 3 days for a total of five injections over 15 days. After 23 days of treatment, cardiac function was assessed by echocardiography. Mice were sacrificed by an overdose of anesthesia, followed by rapid excision and weighing of their hearts. Cardiac tissues were preserved using 4% paraformaldehyde fixation or flash–frozen at −80°C for later analysis and Western blotting. All methods were performed in accordance with the relevant guidelines and regulations and ARRIVE guidelines. Figure 1 depicts the experimental timeline.

### 2.4 Echocardiography

On the Day 23 of the experiment, echocardiography was performed on three randomly selected mice from each group using a Vevo 2100 UBM system (VisualSonics, Canada). Mice were anesthetized with 1%–3% isoflurane in oxygen and maintained under anesthesia throughout the procedure. Key left ventricular functional parameters were assessed, including left ventricular ejection fraction (LVEF), left ventricular fractional shortening (LVFS), left ventricular end‐diastolic diameter (LVEDD), left ventricular end‐systolic diameter (LVESD), left ventricular end‐diastolic volume (LVEDV), left ventricular end‐systolic volume (LVESV), peak systolic velocity of the mitral annulus (S′), peak early diastolic velocity of the mitral annulus (E'), and the ratio of early to late diastolic mitral inflow velocities (E/A ratio). All measurements were calculated using Vevo Analysis software (VisualSonics).

### 2.5 Histological Examination

The mice's hearts were washed with PBS and then fixed in 10% formalin at 4°C overnight. Subsequently, the samples underwent dehydration, infiltration, paraffin embedding, and were sliced into 5 μm‐thick sections using a pathological microtome. The sections were then stained with Hematoxylin and Eosin (HE).

### 2.6 TUNEL Assay

Cardiac apoptosis was assessed using TUNEL staining, following the manufacturer's guidelines (Servicebio, China). Apoptosis was measured by the proportion of TUNEL‐positive nuclei observed in three images per heart.

### 2.7 Evaluation of Marker of Myocardial Injury

Serum CK‐MB levels were measured using commercial kits from Nanjing Jiancheng Bioengineering Institute and Beyotime Institute of Biotechnology, following the manufacturers' guidelines. The experiment concluded with the collection of mouse hearts from each group. The hearts were washed three times with phosphate buffer and then homogenized with a 4:1 ratio of cold phosphate buffer to heart tissue mass. The homogenate was centrifuged at 4000 g for 10 min at 4°C. The total protein in the supernatants was assayed using the BCA protein kit. These supernatants were kept at −80°C for measuring MDA and SOD in heart tissues.

### 2.8 Evaluation of Mitochondrial Function

The mitochondria from heart tissue were extracted, and their activity was measured by assessing the ATP content inside the mitochondria with an ATP kit.

### 2.9 Western Blot

Proteins were isolated and measured from the heart tissues of mice. Samples containing 30 μg of protein were subjected to SDS‐PAGE and then transferred onto PVDF membranes. Membranes were blocked using 5% skim milk and incubated overnight at 4°C with primary antibodies against HDAC8, BRCA2, DRP1, Bcl‐2, and *β*‐actin at a 1:2000 dilution. After being washed three times with TBST, the membranes were exposed to HRP‐linked secondary antibodies (diluted 1:1000) at room temperature for an hour. ECL reagents were used to visualize protein bands, and ImageJ software was employed for semi‐quantification. The density of the target protein was normalized with *β*‐actin for statistical analysis.

### 2.10 Immunofluorescence Staining

The colocalization of HDAC8, BRCA2, and DRP1 within cardiac tissues was evaluated through immunofluorescence staining. Initially, heart tissue sections underwent dewaxing in xylene, followed by rehydration using ethanol. Subsequently, the sections were treated with 3% hydrogen peroxide for 15 min to inhibit endogenous peroxidase activity. Antigen retrieval was performed using a 0.1 M sodium citrate solution. The sections were then blocked with bovine serum albumin (BSA) for 1 h at room temperature, after which they were incubated with primary antibodies specific to HDAC8, BRCA2, and DRP1 at a dilution of 1:200 for an additional hour under the same conditions. Secondary antibodies conjugated with Cy3 and FITC were applied at a 1:200 dilution for 1 h at room temperature. Nuclei underwent a 10‐min staining process using DAPI. Fluorescence imaging was performed using a fluorescence microscope to visualize HDAC8, BRCA2, and DRP1 interactions.

### 2.11 Cell Culture and Treatment

The H9C2 cell line, sourced from the Cell Bank of the Chinese Academy of Sciences in Shanghai, China, was cultured in DMEM supplemented with 10% FBS and 1% penicillin–streptomycin. The cells were grown at 37°C in a humid atmosphere containing 95% air and 5% CO_2_, and they were subcultured every 2–3 days. The cells were split into four experimental groups: (1) Control group, cultured in standard DMEM; (2) DOX group, incubated in standard medium for 24 h, then exposed to 1 μM DOX for another 24 h; (3) TSF group, pretreated with TSF at concentrations of 0.5, 2.5, 5, 10, 25, or 50 μg/mL for 24 h, followed by 24‐h exposure to 1 μM DOX; (4) Rg3 group, pretreated with Rg3 at concentrations of 0.1, 1, 10, or 50 μg/mL for 24 h, followed by 24‐h exposure to 1 μM DOX. Following treatment, cell viability was quantified via the Cell Counting Kit‐8 (CCK‐8) assay. Cell survival rate was calculated using the formula: Cell survival rate = ([OD value of treated group−OD value of blank group]/[OD value of control group −OD value of blank group]) × 100%. Here, the blank group refers to the well containing only the culture medium without cells.

### 2.12 Data Analysis

The data are shown as the mean ± standard. Group comparisons were evaluated using SPSS 25 for ANOVA. GraphPad Prism (version 8.02) was utilized for plotting the data.

### 2.13 PCA Analysis

PCA was performed to conduct dimensionality reduction and clustering analysis on the core indicators reflecting the severity and pathological status of HF, including MDA, SOD, CK‐MB, and ATP. The PCA results were visualized as a score plot, in which each discrete data point corresponded to an individual biological sample.

## 3 Results

### 3.1 TSF Mitigates DOX‐Induced Cardiac Damage by Modulating the HDAC8/BRCA2/DRP1 Pathway

**FIGURE 2:**
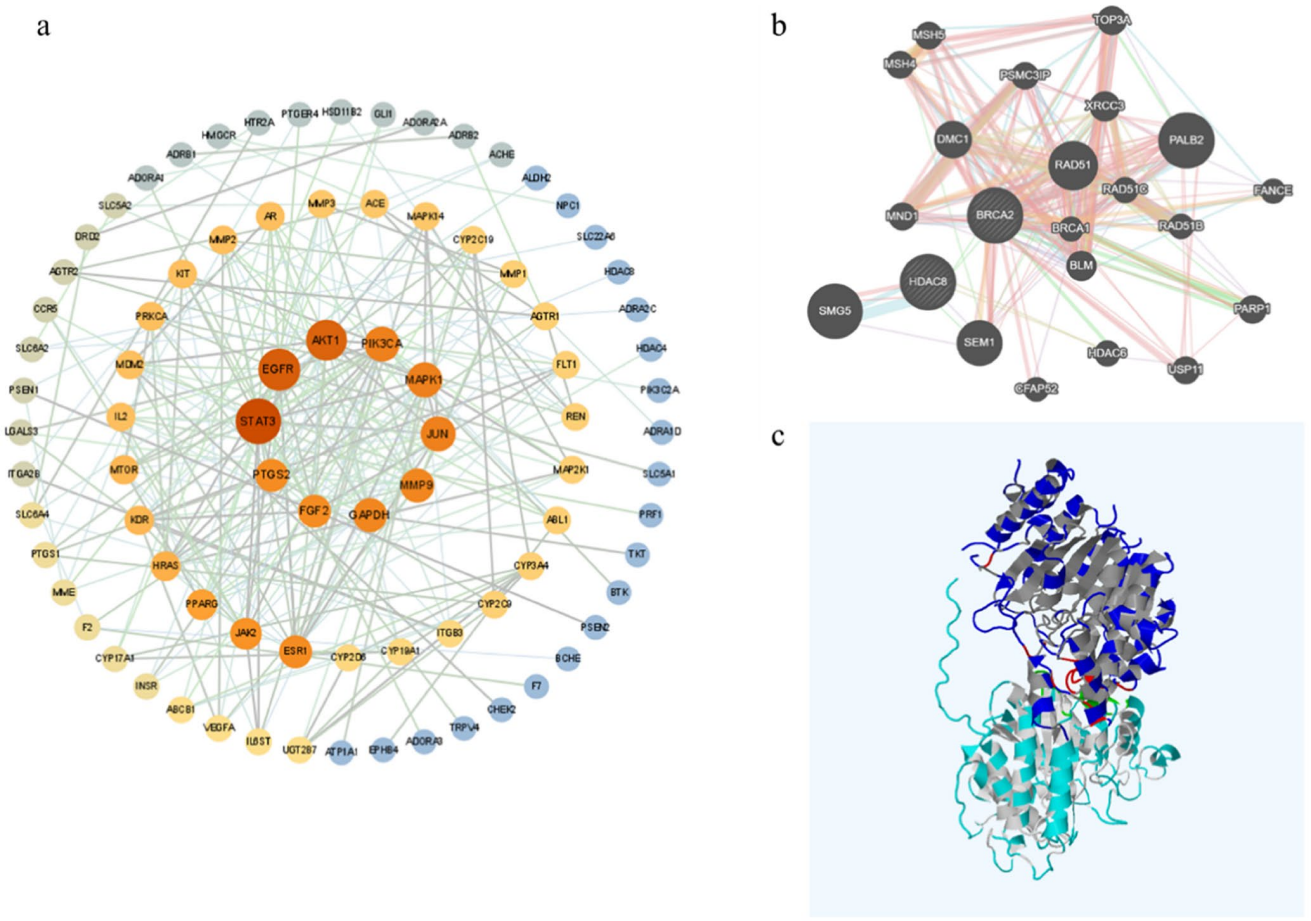
The potential function and pathways of TSF on DOX‐induced cardiotoxicity: (a) PPI network of 103 overlapping targets constructed based on the STRING database; (b) the PPI network map; (c) the molecular docking of HDAC8 with BRCA2.

Based on the results obtained from the TCMSP search, 30 active ingredients of TSF were identified following screening, corresponding to 1038 potential targets (refer to Supporting Information S2). The GeneCards, OMIM, and TTD databases were utilized to independently query 13,391, 140, and 75 targets associated with HF disease, respectively. Utilizing the Venn Diagram tool in R, a total of 103 intersecting genes between TSF and HF were identified. A protein–protein interaction (PPI) network was then created by submitting the overlapping targets to the STRING database. The findings indicate that HDAC8 and other key targets for treating DOX‐induced HF with TSF have been identified, with an interaction observed between HDAC8 and BRCA2 (Figure 2a,b). Molecular docking analyses of the targets HDAC8 and BRCA2 are provided (Figure 2c). Analysis using the PhosphoSite database revealed the presence of acetylation sites on BRCA2. Furthermore, the RNAct database predicted that BRCA2 possesses an RNA‐binding domain capable of strongly binding to the DRP1 mRNA sequence. Based on these findings, we infer that TSF may ultimately improve the progression of DOX‐induced HF by targeting and regulating the HDAC8/BRCA2/DRP1 pathway.

### 3.2 TSF and Rg3 Alleviates DOX‐Induced Myocardial Injury

**FIGURE 3:**
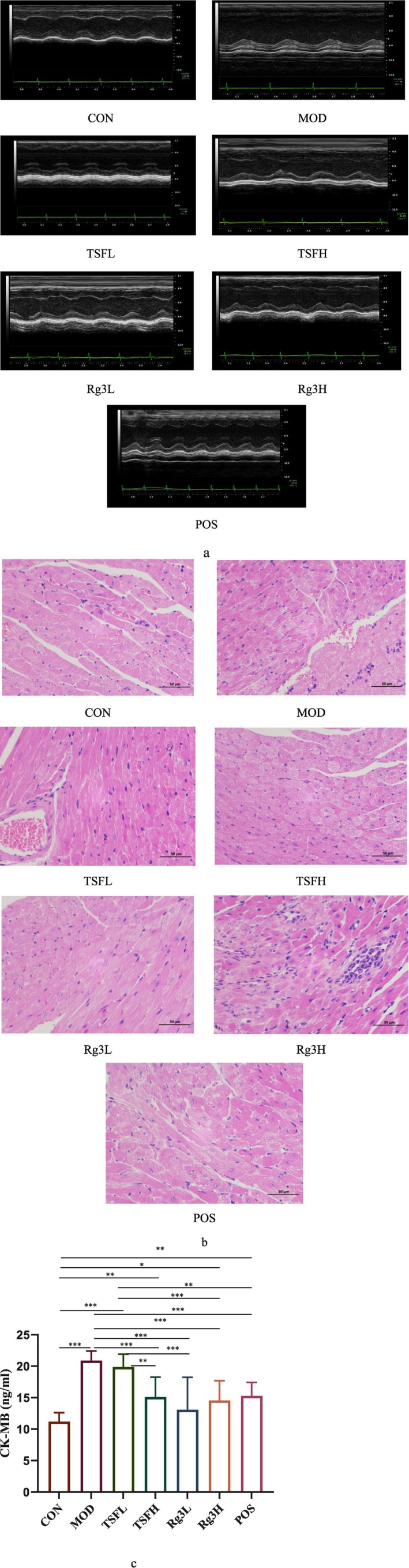
TSF and Rg3 alleviates DOX‐induced myocardial injury (a) Alterations in high‐frequency echocardiography in mouse heart tissue. (b) HE staining of heart tissues (Scale bar = 50 μm, magnification ×200). (c) The changes in the content of CK‐MB in each group. Data were collected from eight mice per group and are presented as the mean and standard. Data analysis among groups was performed using ANOVA, with LSD for equal variances and Dunnett's‐T3 for unequal variances, with significance levels of *p < 0.05, **p < 0.01, ***p < 0.001.

Echocardiographic analysis revealed that mice in the MOD group showed a significant reduction in left ventricular ejection fraction and fractional shortening compared to the CON group. Notably, DOX‐treated mice receiving both high and low doses of TSF and Rg3 exhibited significant improvements in cardiac parameters, as shown in Figure 3a. HE stained sections showed typical myofibrillar structures in the CON group. In contrast, the MOD group displayed myocardial degeneration, irregular myofibrils, and cytoplasmic vacuolization across numerous myocardial areas. Figure 3b demonstrates that both high and low doses of TSF and Rg3 effectively reduced myocardial damage. Figure 3c shows that the MOD group had higher CK‐MB levels than the CON group (*F* = 11.46, *p* = 0.001), while the TSFH, Rg3L, Rg3H, and POS groups demonstrated significantly reduced CK‐MB concentrations compared to the MOD group (TSFH: *F* = 11.46, *p* = 0.001; Rg3L: *F* = 11.46, *p* = 0.001; Rg3H: *F* = 11.46, *p* = 0.001; POS: *F* = 11.46, *p* = 0.001).

### 3.3 TSF and Rg3 Reduces DOX‐Induced Oxidative Stress in the Heart

**FIGURE 4:**
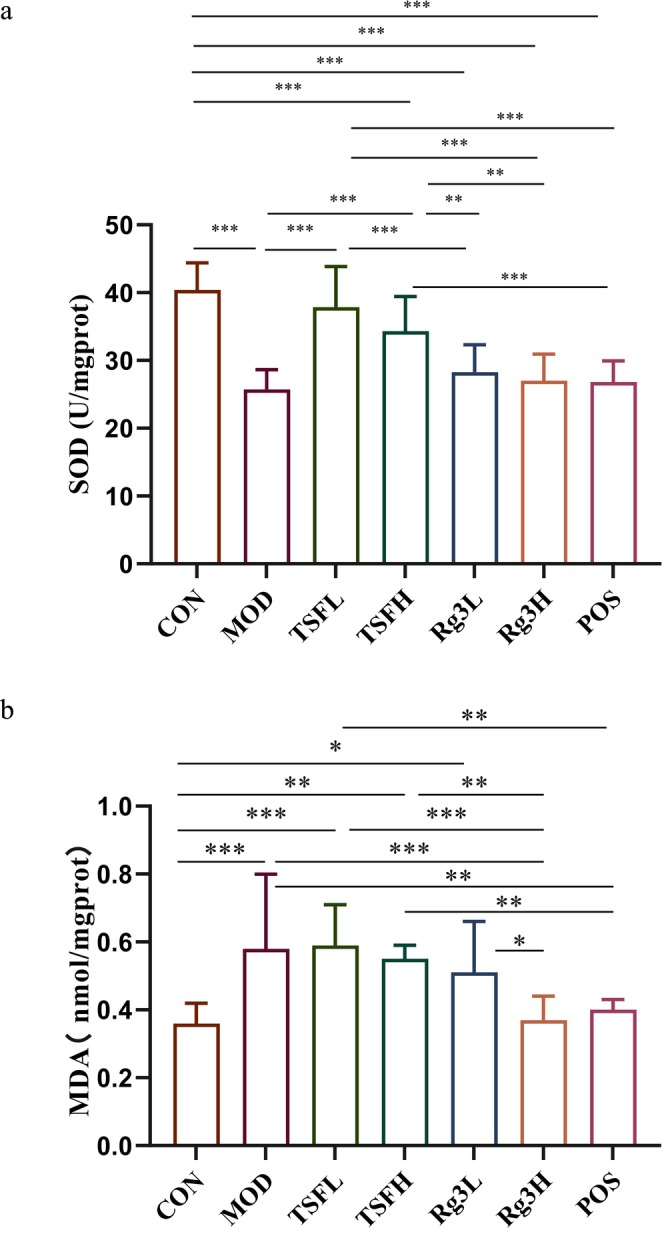
TSF and Rg3 alleviates DOX‐induced myocardial injury. (a) The changes in the activity of SOD in each group. (b) The changes in the activity of MDA in each group. Data were collected from eight mice per group and are presented as the mean and standard. Data analysis among groups was performed using ANOVA, with LSD for equal variances and Dunnett's‐T3 for unequal variances, with significance levels of *p < 0.05, **p < 0.01, ***p < 0.001.

Figure 4a shows a significant reduction in SOD activity in the MOD group compared to the CON group (*F* = 15.54, *p* = 0.001). Conversely, the SOD activity in the other treatment groups demonstrated an upward trend, with the TSFL and TSFH groups exhibiting significantly higher activity than the MOD group (TSFL: *F* = 15.54, *p* = 0.001; TSFH: *F* = 15.54, *p* = 0.001). Figure 4b depicts the alterations in MDA activity. The MOD group displayed significantly elevated MDA activity relative to the CON group (*F* = 5.893, *p* = 0.001), whereas the Rg3H group showed significantly reduced activity compared to the MOD group (*F* = 5.893, *p* = 0.001). These findings suggest that TSF and Rg3 have the potential to mitigate DOX‐induced oxidative stress.

### 3.4 TSF and Rg3 Reversed DOX‐Induced Mitochondrial Damage in Mice

**FIGURE 5:**
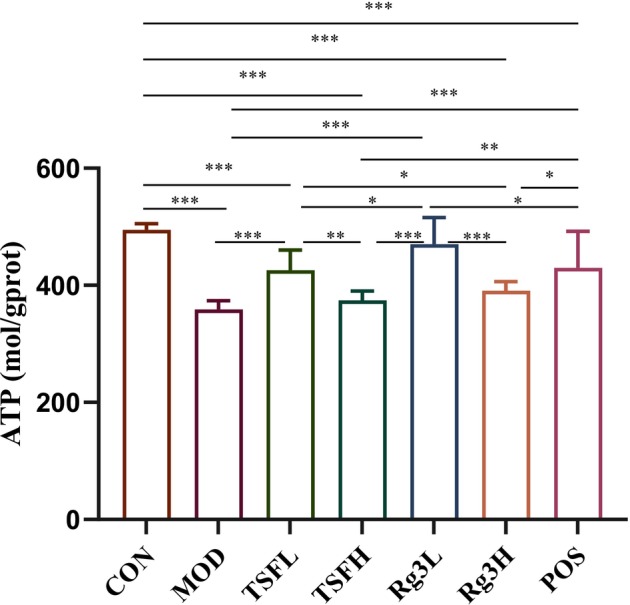
The changes in the activity of ATP in each group. Data were collected from eight mice per group and are presented as the mean and standard deviation. Data analysis among groups was performed using ANOVA, with LSD for equal variances and Dunnett’s‐T3 for unequal variances, with significance levels of *p < 0.05, **p < 0.01, ***p < 0.001.

Mitochondria play a crucial role in cellular energy metabolism (Kong et al. 2025). Dysfunction in mitochondrial processes frequently results in impaired ATP production and activity, leading to disruptions in energy metabolism, as depicted in Figure 5. In the MOD group, myocardial ATP activity was markedly diminished (*F* = 17.499, *p* = 0.001), indicating compromised energy metabolism. Treatment with TSFL, Rg3L, and POS successfully restored these levels (TSFL: *F* = 17.499, *p* = 0.001; Rg3L: *F* = 17.499, *p* = 0.001; POS: *F* = 17.499, *p* = 0.001), underscoring their role in enhancing mitochondrial function and energy homeostasis. In conclusion, TSFL and Rg3 demonstrate efficacy in mitigating DOX‐induced mitochondrial damage, facilitating the recovery of energy metabolism, and contributing to the maintenance of cardiac function.

### 3.5 TSF and Rg3 Regulates HDAC8/BRCA2/DRP1 Pathway Inhibits DOX‐Induced Cardiomyocyte Apoptosis

**FIGURE 6:**
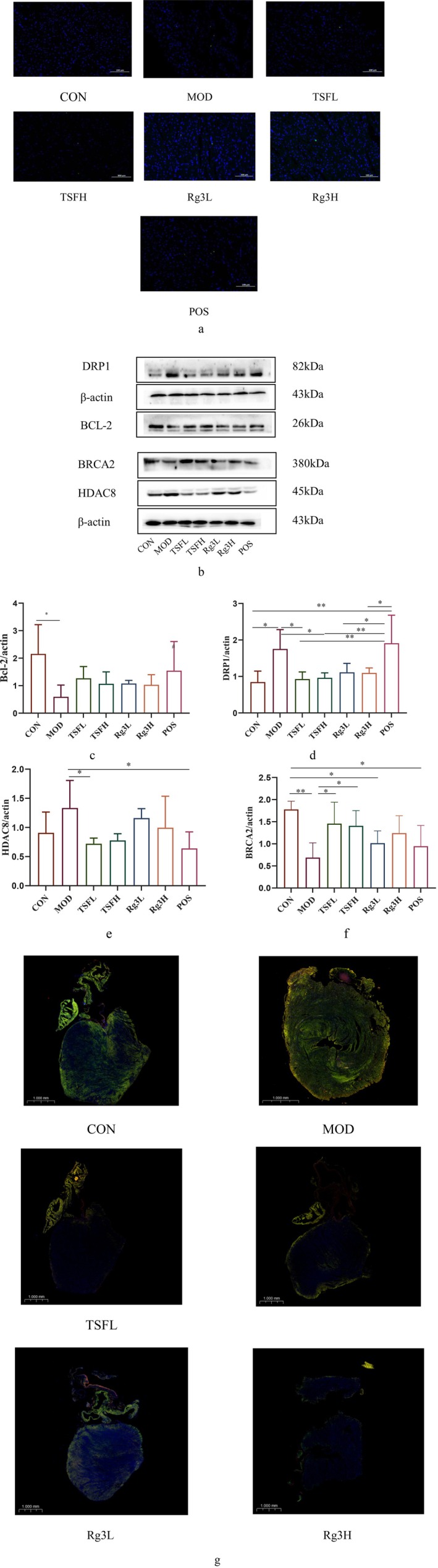
TSF and Rg3 regulate HDAC8/BRCA2/DRP1 pathway inhibits DOX‐induced cardiomyocyte apoptosis. (a) Apoptosis was assessed using TUNEL staining (Scale bar = 50 μm). (b) The results of western blotting for each group. (c) The expression of Bcl‐2 for each group. (d) The expression of DRP1 for each group. (e) The expression of HDAC8 for each group. (f) The expression of BRCA2 for each group. (g) Immunofluorescence staining for observing the colocalization of HDAC8, BRCA2, and DRP1 (Scale bar = 1 mm). Data are expressed as mean ± standard from three individual experiments. Data analysis among groups was performed using ANOVA, with LSD for equal variances and Dunnett’s‐T3 for unequal variances, with significance levels of *p < 0.05, **p < 0.01, ***p < 0.001.

TUNEL staining was employed to assess the impact of DOX on cardiomyocyte apoptosis. The findings revealed a notable rise in TUNEL‐positive cells in the MOD group relative to the control group. Figure 6a demonstrates a significant reduction in this number in groups treated with both high and low doses of TSF and Rg3 compared to the MOD group. Western blot analysis indicated that DOX treatment decreased Bcl‐2 expression (*F* = 1.718, *p* = 0.011). In comparison to the model group, the Bcl‐2/actin ratio exhibited an increasing trend in each treatment group, as shown in Figure 6b,c.

Figure 6d–f demonstrate that the HDAC8/actin and DRP1/actin ratios were higher in the MOD group than in the CON group, with a statistically significant increase observed in the DRP1/actin ratio (HDAC8/actin: *F* = 1.688, *p* = 0.137; DRP1/actin: *F* = 3.447, *p* = 0.014). Conversely, the BRCA2/actin ratio exhibited a significant decrease (*F* = 2.956, *p* = 0.003). The HDAC8/actin ratio was significantly lower in both the TSFL group and the POS group compared to the MOD group (TSFL: *F* = 1.688, *p* = 0040; POS: *F* = 3.447, *p* = 0.023). Additionally, the DRP1/actin ratio significantly decreased in both the high and low‐dose TSF administration groups (TSFL: *F* = 3.447, *p* = 0.023; TSFH: F = 3.447, *p* = 0.028), while the BRCA2/actin ratio significantly increased in these groups (TSFL: *F* = 2.956, *p* = 0.023; TSFH: *F* = 2.956, *p* = 0.032). To explore the interactions among HDAC8, BRCA2, and DRP1, immunofluorescent staining was performed on mouse heart tissues. As depicted in Figure 6g, the fluorescence intensities of HDAC8 (red light) and DRP1 (yellow light) were enhanced in the heart tissues of the MOD group compared to the CON group. In contrast, these fluorescence intensities were reduced in the high and low‐dose TSF groups compared to the MOD group. Furthermore, the fluorescence intensity of BRCA2 (green light) was enhanced in the low‐dose TSF group. These results showed that TSF and Rg3 modulate the HDAC8/BRCA2/DRP1 pathway, reducing DOX‐induced apoptosis in cardiomyocytes.

### 3.6 TSF and Rg3 Improve the Survival Rate of H9c2 Cells Impacted by DOX

**FIGURE 7:**
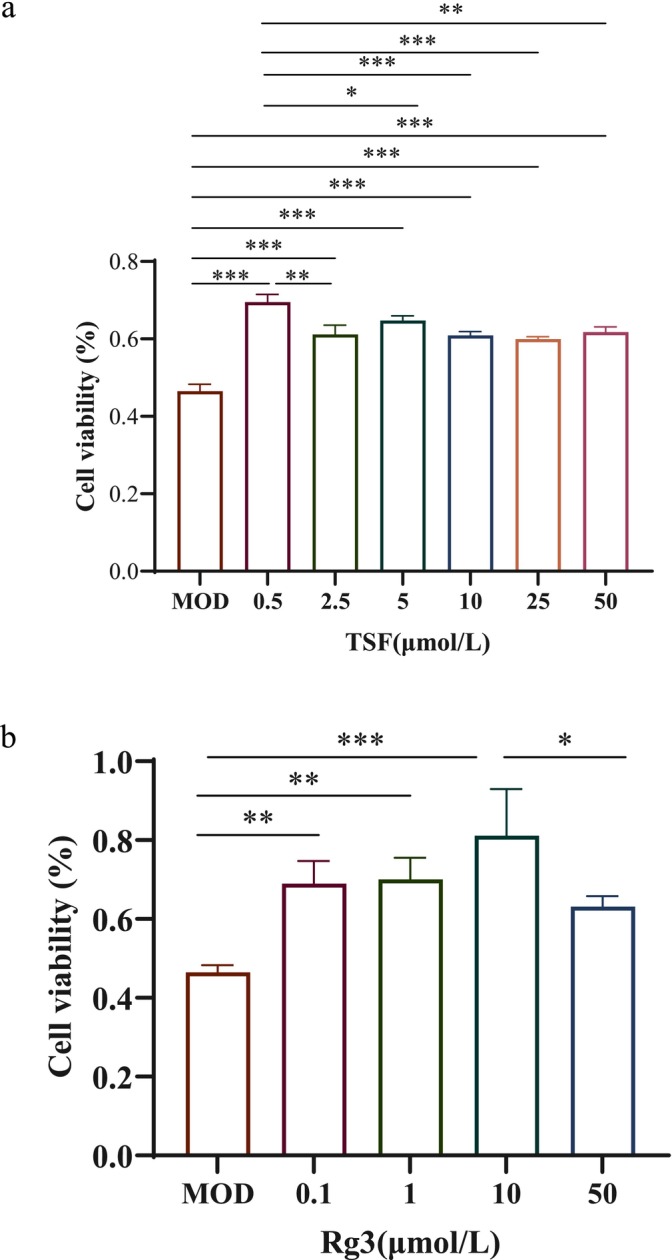
TSF and Rg3 can enhance the survival rate of H9c2 cells affected by DOX. (a) The influence of TSF at various doses on the survival rate of H9C2 cells. (b) The influence of Rg3 at various doses on the survival rate of H9C2 cells. Data are expressed as mean ± standard from three individual experiments. Data analysis among groups was performed using ANOVA, with LSD for equal variances and Dunnett’s‐T3 for unequal variances, with significance levels of *p < 0.05, **p < 0.01, ***p < 0.001.

As illustrated in Figure 7, both TSF and Rg3 exhibit protective effects on H9C2 cells. TSF is most effective at 0.5 μmol/L (F = 39.363, *p* = 0.001), while Rg3 performs best at 10 μmol/L (F = 7.532, *p* = 0.003).

### 3.7 Comparative Analysis of Treatment Group Efficacy

**FIGURE 8:**
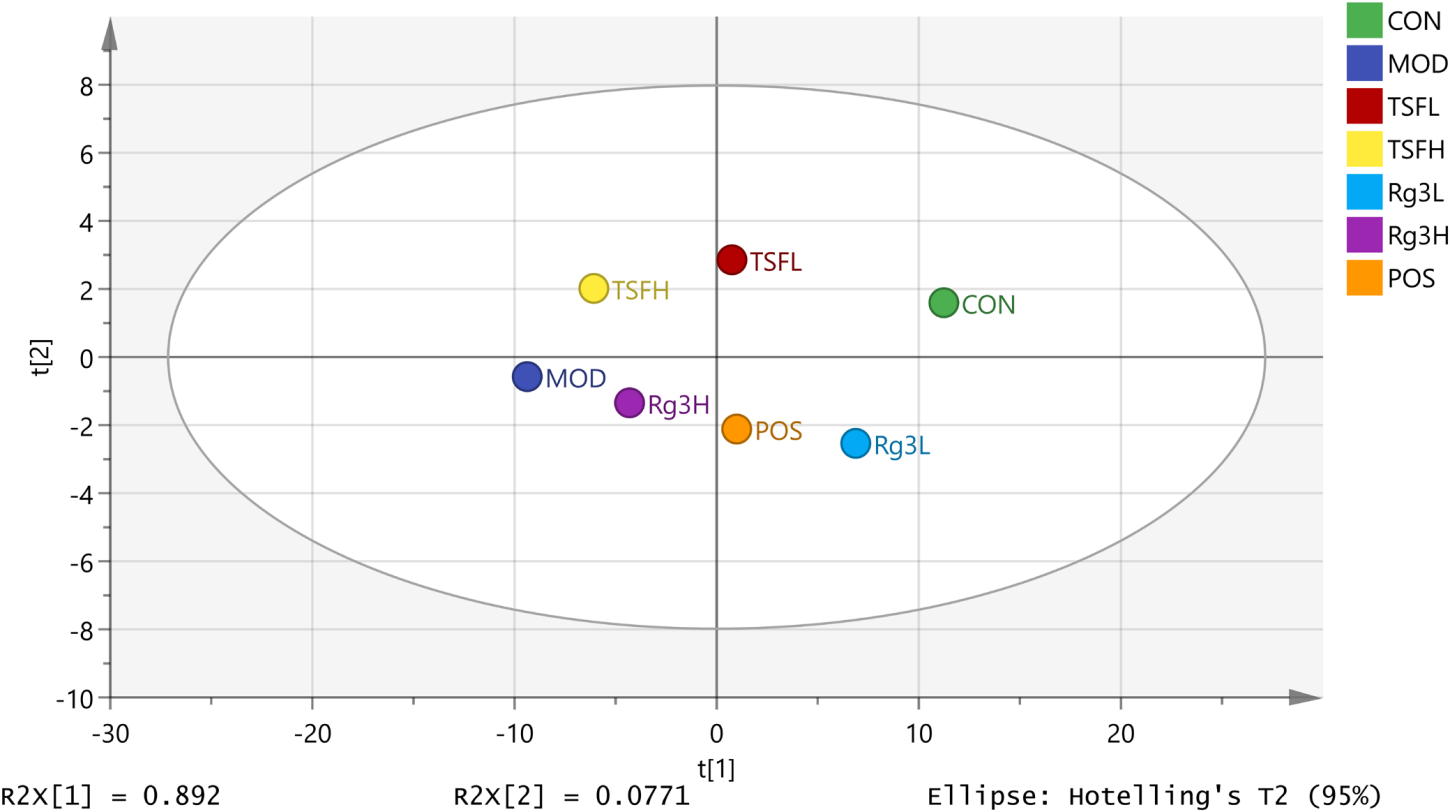
Analysis of PCA scores plots.

As presented in Figure 8, PCA indicated that the high and low‐dose Rg3 groups were positioned much closer to the CON group compared with the high and low‐dose TSF groups. This clustering trend provides clear evidence that Rg3 exerted a more prominent biological effect under the current experimental conditions.

## 4 Discussion

Ginseng, specifically the dried root and rhizome of *Panax ginseng* C. A. Mey., is a member of the Panax genus within the Araliaceae family. Traditionally, it is known for replenishing vital energy, strengthening the pulse, stopping hemorrhage, and nourishing the spleen, lungs, fluids, and blood. BG is derived through microbial fermentation or repeated steaming and drying processes of ginseng, resulting in substantial alterations in its chemical composition and pharmacological properties. BG has been industrialized in Liaoning Province. Previous research conducted by the team revealed that the trans‐formation of ginseng into BG leads to changes in chemical components and concentrations, including saponins, amino acids, and oligosaccharides, with a notably high content of rare saponins in BG. The alteration in chemical components and contents may be associated with an enhanced pharmacological effect. In the context of TCM, HF is categorized under heart water and heart palpitations, with its pathogenesis attributed to deficiency of heart *qi*, *cold* yang, and blood stasis. A deficiency in heart *qi* results in impaired blood circulation and obstructed blood vessels, leading to symptoms such as palpitations and a weak pulse. GS's reputed ability to greatly replenish vital energy is believed to address the deficiency of heart qi, while its properties of reinforcing the pulse and stopping hemorrhage are thought to restore the smooth flow of the pulse pathway. This aligns with the pathological process of qi deficiency causing blood stasis observed in HF. Building upon the theoretical correlation, the research team previously identified the HF model characterized by heart *qi* deficiency as the pathological foundation for their study. They conducted a systematic comparison of the effects of GS, RG, and BG on energy metabolism, substance metabolism, and the nervous and endocrine systems. The findings revealed distinct mechanisms of action for each of the three substances. Notably, BG demonstrated a significant capacity to enhance myocardial energy supply in HF model Mice, exhibiting a potent anti‐HF effect. However, the precise mechanisms underlying the increased efficacy of BG through processing remain inadequately elucidated.

Cyberpharmacology leverages databases like proteomics, genomics, and bioinformatics to enhance research in Chinese medicine. This approach allows for a comprehensive analysis of Chinese medicine at molecular and holistic levels, aiding in the identification of key chemical components and protein targets in traditional Chinese medicine (TCM) and clarifying its mechanisms of action (Jiang et al. 2022). The research group's network pharmacology analysis identified HDAC8 as a key target for BG in improving HF. HDAC8, part of the histone deacetylase family, regulates the deacetylation of histone and nonhistone proteins. Research indicates that suppressing HDAC8 expression improves HF in mice with aortic stenosis (Rajaraman et al. 2023), while its activation worsens mitochondrial dysfunction in cardiomyocytes (Lkhagva et al. 2018). Further investigations suggest that HDAC8 may modulate the expression of BRCA2 through deacetylation modification. Literature indicates that BRCA2 deficiency is linked to heightened HF susceptibility and mitochondrial oxidative stress induction (Renaudin et al. 2021; Singh et al. 2012). Furthermore, BRCA2 possesses an RNA‐binding domain that exhibits a high affinity for the mRNA sequence of DRP1. Research indicates that DRP1 is upregulated in murine HF models, disrupting mitochondrial homeostasis, and causing dysfunction, which worsens cardiac toxicity (Zhuang et al. 2021). The results indicate that TSF could alleviate HF by modulating the HDAC8/BRCA2/DRP1 pathway to reduce mitochondrial dysfunction.

**FIGURE 9:**
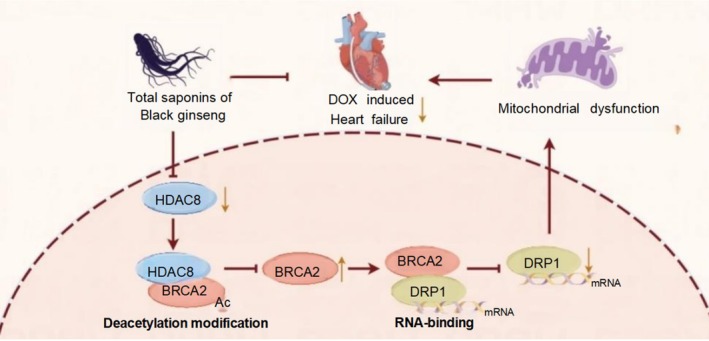
The mechanism diagram of TSF and Rg3 alleviating DOX‐induced myocardial injury by regulating the HDAC8/BRCA2/DRP1 pathway.

This research investigated the cardioprotective effects of TSF and Rg3 against DOX‐induced myocardial damage using both in vivo and in vitro models. A 1 μM concentration of DOX significantly reduced H9c2 cell viability, indicating cellular damage. The MOD group received intraperitoneal DOX at 2.5 mg/kg every 3 days, totaling five injections and a cumulative dose of 12.5 mg/kg. Mice in the MOD group showed weight loss, elevated respiratory rates, reduced activity, dull fur, lethargy, and delayed responses compared to the CON group. Histological examination with HE staining showed tissue infiltration by inflammatory cells. Furthermore, CK‐MB levels were elevated. Echocardiographic assessments demonstrated a reduction in LVEF and LVFS, along with an increase in LVIDs. The indices and histological findings corroborate previous research, confirming the successful replication of DIC in cellular and murine models (Hu et al. 2022; Xu et al. 2022). In both in vitro and in vivo studies, TSF and Rg3 administration notably improved myocardial dysfunction in DOX models. Notably, the effect of Rg3 was particularly pronounced. The treatment effectively inhibited key DOX‐associated biomarkers, such as CK‐MB, and mitigated oxidative stress markers, including SOD and MDA, as well as inflammation. Furthermore, it prevented apoptosis, improved cardiomyocyte structure, and boosted mitochondrial function by elevating ATP activity. Fluorescence staining analysis indicated that the HDAC8/BRCA2/DRP1 signaling pathway may serve as a target for the regulation by TSF and Rg3 during DOX‐induced progression. Our findings demonstrated that TSF and Rg3 reverse DOX‐induced upregulation of HDAC8 and DRP1 protein expression while downregulating BRCA2 protein expression in cardiac tissues. Furthermore, immunofluorescence colocalization confirmed direct binding among HDAC8, BRCA2, and DRP1. These findings indicate that TSF and Rg3 alleviate DOX‐induced myocardial injury through modulation of the HDAC8/BRCA2/DRP1 signaling pathway, as illustrated in Figure 9. The study has presented new therapeutic approaches and targets for the clinical management of myocardial damage due to DOX.

## 5 Conclusion

Our findings indicate that TSF and Rg3 effectively mitigate DOX‐induced oxidative stress, inflammation, apoptosis, and mitochondrial dysfunction, leading to improved cardiac function. Preliminary evidence suggests that TSF and Rg3 reduce DOX‐induced cardiomyocyte apoptosis through a multi‐target, multi‐pathway approach, partly by influencing the HDAC8/BRCA2/DRP1 pathway to reverse mitochondrial damage and alleviate DOX‐related pathological symptoms. Our study examined the HDAC8/BRCA2/DRP1 pathway, an underexplored but promising target. This pathway is essential for energy metabolism, cellular stress response, and maintaining mitochondrial homeostasis. A thorough examination of this signaling axis is anticipated to offer new insights and a theoretical basis for further understanding the pathogenesis of DOX‐induced myocardial injury. This may provide new insights and research directions for comprehensively understanding the cardioprotective mechanisms of TSF and Rg3. The study lacked the use of HDAC8/BRCA2/DRP1 pathway‐related activators, inhibitors, or gene knockout mice for mechanism validation. This limitation should be addressed in future research. However, HDAC8/BRCA2/DRP1 pathway‐related activators, inhibitors, or gene knockout mice were not used here to validate its mechanism. Future research should investigate the regulatory role of this pathway using specific agonists/inhibitors and genetic manipulation in vitro and in vivo.

## Author Contributions

**Linlin Liu:** designed experiments, conduct experiments, analyze experimental data, and wrote the manuscript. **Peiyuan Dou:** design experiments, analyze experimental data. **Xiaotong Zhang:** methodology. **Xiaoku Ran and Xiaotong Zhang:** participate in animal experiments and Elisas determination. **Deqiang Dou:** conceive and supervise research, coordinate technical support and project funding. **Xiaodong Lv:** supervision, writing – original draft. All authors have read and approved the final manuscript.

## Funding

This research was granted by Liaoning Province Applied Basic Research Program (grant no. 2025JH2/101330066) and the Research Project for Universities of Liaoning Provincial Education Department (grant no. LJ222510162006).

## Conflicts of Interest

The authors declare no conflicts of interest.

## Supporting information


**Supporting Information S1.** Compounds of TSF analyzed by HPLC.
**Supporting Information S2.** Active ingredients of TSF.

## Data Availability

The data supporting the results of this study can be obtained from the methods or supporting information in this article.
