# Supplementary material for: Exploring the Mechanisms of Total Saponins of Black Ginseng and Ginsenoside Rg3 Against Doxorubicin‐Induced Cardiotoxicity
Source: Food Sci Nutr. 2026 Jun 8;14(6):e71968. doi: 10.1002/fsn3.71968 (PMC13247116; doi:10.1002/fsn3.71968)
Supplement: Supplementary file 1 — Supporting Information S1. Compounds of TSF analyzed by HPLC. Supporting Information S2. Active ingredients of TSF. [file FSN3-14-e71968-s001.docx]

**Supplemental Material**

S1. Compounds of TSF analyzed by HPLC


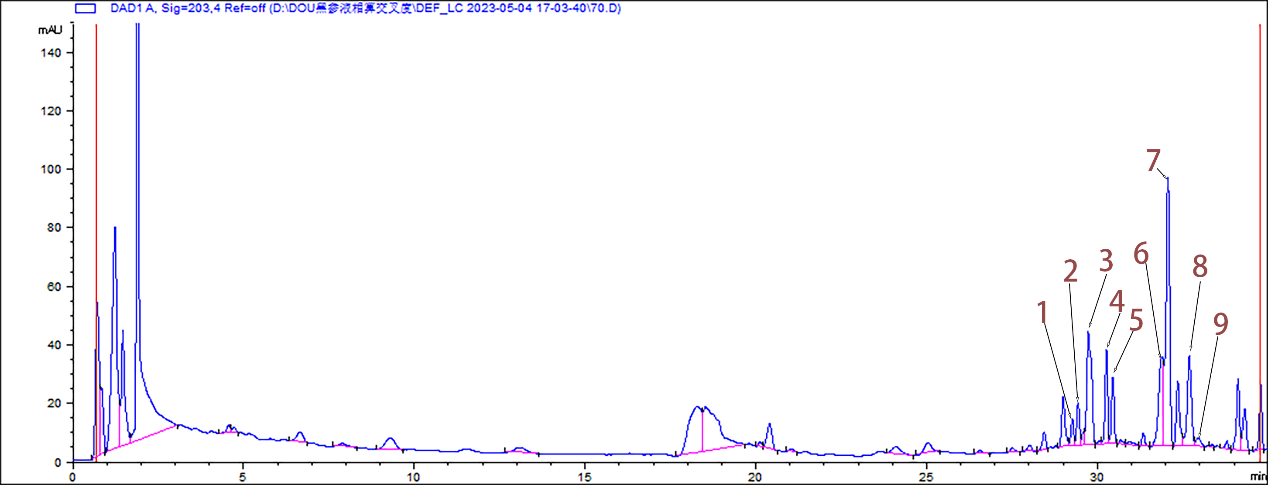


Note：1. F4；2. Rk3；3. Rh4；4.20 (S) -Rg3；5. 20 (R) - Rg3 ；6. Rk1；7. Rg5；8.20 (R)-Rh；9.20-(R)-Rh2

S2. Active ingredients of TSF

| NO. | Compound Name | Gene symbol Number | NO. | Compound Name | Gene symbol Number |
| --- | --- | --- | --- | --- | --- |
| 1 | Ginsenoside Compound K | 24 | 16 | Ginsenoside Rg3 | 22 |
| 2 | Ginsenoside Compound Y | 25 | 17 | Ginsenoside Rg4 | 31 |
| 3 | Ginsenoside F1 | 25 | 18 | Ginsenoside Rg5 | 48 |
| 4 | Ginsenoside F2 | 20 | 19 | Ginsenoside Rg6 | 18 |
| 5 | Ginsenoside F4 | 42 | 20 | Ginsenoside Rh1 | 33 |
| 6 | Ginsenoside Mc | 20 | 21 | Ginsenoside Rh2 | 25 |
| 7 | Ginsenoside Rb1 | 23 | 22 | Ginsenoside Rh3 | 114 |
| 8 | Ginsenoside Rb2 | 23 | 23 | Ginsenoside Rh4 | 102 |
| 9 | Ginsenoside Rb3 | 23 | 24 | Ginsenoside Rk1 | 32 |
| 10 | Ginsenoside Rc | 45 | 25 | Ginsenoside Rk2 | 114 |
| 11 | Ginsenoside Rd | 23 | 26 | Ginsenoside Rk3 | 94 |
| 12 | Ginsenoside Re | 22 | 27 | Ginsenoside Ro | 19 |
| 13 | Ginsenoside Rf | 20 | 28 | Ginsenoside Rs1 | 21 |
| 14 | Ginsenoside Rg1 | 24 | 29 | Ginsenoside Rs2 | 20 |
| 15 | Ginsenoside Rg2 | 10 | 30 | Ginsenoside Rs3 | 21 |
